# A Multicentre, Prospective, Non‐Interventional Single‐Arm Study Investigating the Impact of Once‐Daily Oral Semaglutide in a Real‐World Adult Population With Type 2 Diabetes in Mexico

**DOI:** 10.1002/edm2.70219

**Published:** 2026-04-15

**Authors:** Guillermo González‐Gálvez, Juan C. Garnica‐Cuellar, Miguel Ángel Colín‐García, Aldo Ferreira‐Hermosillo, Silvia A. Jiménez‐Ramos, Aleida Y. Contreras‐Sandoval, Patricia Cruz‐Puente, Manuel Duarte‐Vega

**Affiliations:** ^1^ Instituto Jalisciense de Investigación en Diabetes y Obesidad Guadalajara Mexico; ^2^ Endocrinology Division Centro Médico Nacional 20 de Noviembre, Institute for Social Security and Services for State Mexico City Mexico; ^3^ Diabetes and Obesity Workgroup Sociedad Mexicana de Nutrición y Endocrinología Mexico City Mexico; ^4^ Colegio Mexicano de Obesidad y Nutrición A.C. José María Rico Mexico City Mexico; ^5^ Hospital Ángeles Santa Mónica Mexico City Mexico; ^6^ Unidad de Investigación Médica en Enfermedades Endocrinas, Centro Médico Nacional Siglo XXI, IMSS Mexico City Mexico; ^7^ CICEJ Centro de Investigación Clínica Endocrinológica de Jalisco Guadalajara Mexico; ^8^ Novo Nordisk Mexico Mexico City Mexico; ^9^ División de Medicina Interna, Hospital Civil Juan I. Menchaca Guadalajara Mexico

**Keywords:** diabetes treatment satisfaction, glycaemic control, Mexico, oral semaglutide, real‐world evidence, type 2 diabetes, weight loss

## Abstract

**Background:**

To investigate the oral semaglutide use among Mexican adults with type 2 diabetes.

**Methods:**

PIONEER REAL was a multinational, open‐label study evaluating oral semaglutide in patients without prior injectable glucose‐lowering treatment. This analysis focuses on data from Mexico.

**Results:**

Of 177 patients, 139 (78.5%) completed the study and 111 (62.7%) remained on treatment at the end. The mean (SD) age was 54.0 (10.96) years, 57.6% were women, 59.3% had obesity, and 44.6% had a medical history of cardiovascular disease. The average T2D duration was 5.7 years (SD 6.45), with mean HbA1c at 8.1% (SD 1.85). At week 38, mean HbA1c had decreased to 6.8% (estimated mean [SE] change in HbA1c, −1.3% [0.11]; *p* < 0.0001), and body weight had decreased from 87.8 kg (19.34) to 85.3 kg (estimated mean [SE] change in body weight, −4.3 kg [0.56]; *p* < 0.0001), as had waist circumference (estimated mean [SE] change, −3.7 cm [0.54]; *p* < 0.0001). Additionally, satisfaction with treatment increased over time. Only 6.2% of patients had adverse events leading to withdrawal of semaglutide.

**Conclusions:**

According to this real‐world study, starting oral semaglutide treatment in Mexico is associated with notable decreases in both HbA1c levels and body weight among patients with type 2 diabetes. Satisfaction with treatment also improved significantly. Moreover, the safety profile is consistent with that of previous studies.

**Trial Registration:** ClinicalTrials.gov registration NCT04601753.

## Graphical abstract

Initiation of oral semaglutide in patients with T2D in routine clinical practice in Mexico significantly reduces HbA1c and weight and improves satisfaction with treatment.

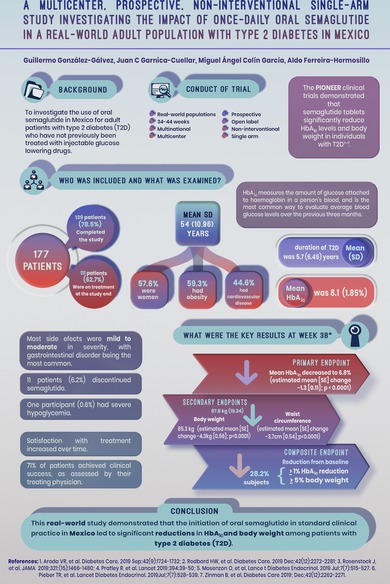

## 1 Background

Type 2 diabetes (T2D) represents a significant health issue in Mexico [1]. In fact, changes in dietary and physical activity patterns in Mexico have led to a significant increase in the prevalence of obesity and diabetes in recent decades [2]. In 2021, Mexico had 14.1 million people with diabetes, with 47.5% undiagnosed. By 2045, cases are expected to rise to 21.2 million. As a result, nearly 17% of adults currently have diabetes. Moreover, the health care costs associated with diabetes are huge in Mexico, with diabetes‐related expenditure in 2021 reaching US$19.9 billion in adults (20–79 years) [3].

Current guidelines recommend a patient‐centred approach to T2D, with the aim not only of achieving adequate glycaemic control, but also comprehensive management of all cardiovascular risk factors and comorbidities to reduce the burden of diabetes [4, 5]. Unfortunately, less than one‐third of patients in Mexico achieve recommended glycaemic targets [1]. Glucagon‐like peptide‐1 receptor agonists (GLP‐1 RAs) are advised for managing hyperglycaemia in T2D, as they help with metabolic control and lower cardiovascular risk, especially for high‐risk patients, regardless of their HbA1c levels [4, 5].

The PIONEER phase 3a clinical trial evaluated how effective and safe oral semaglutide is in over 9500 patients with T2D (with an average disease duration between 3.5 and 15 years). These patients were on various background treatments, including monotherapy, combinations with one or two oral glucose‐lowering agents, or combinations with insulin. These studies evaluated oral semaglutide at doses of 3 mg, 7 mg, and 14 mg in comparison to placebo or active comparators, including empagliflozin 25 mg, sitagliptin 100 mg, and liraglutide 1.8 mg (Supplementary Table 1) [6, 7, 8, 9, 10, 11, 12, 13]. In PIONEER 2, oral semaglutide 14 mg daily reduced HbA1c and body weight more than empagliflozin 25 mg over 52 weeks in 822 T2D patients on metformin [7]. The PIONEER 3 study found that, among 1864 patients with T2D who were not adequately managed with metformin (with or without sulfonylurea), taking oral semaglutide at doses of 7 mg/day or 14 mg/day led to significantly greater reductions in HbA1c and body weight compared to sitagliptin after 26 weeks of treatment [8]. PIONEER 6, which included 3183 patients with T2D at elevated cardiovascular risk, demonstrated that treatment with oral semaglutide 14 mg per day was associated with a tendency toward reduced risk of major adverse cardiovascular events, as well as lower rates of both cardiovascular and all‐cause mortality, following a median follow‐up period of 69 weeks [11].

Clinical trials are the gold standard for evaluating treatment efficacy and safety, but their patient populations are more selective and closely monitored than those in real‐world settings. In this context, real‐world evidence studies can better reflect how a drug works in clinical practice, including effectiveness, safety, quality of life, and satisfaction with treatment. Moreover, non‐interventional studies provide relevant information in the general population, albeit in specific risk populations, such as elderly, fragile patients or patients with specific conditions. As a result, real‐world evidence provides important information that complements and completes data from clinical trials and interventional studies, making it more representative of patients undergoing treatment in daily clinical practice [14, 15].

Real‐world data on the management of patients with T2D, including treatment with semaglutide, are very scarce, with the result that specific information is warranted [1, 2, 3]. The PIONEER REAL trial, which was initiated by Novo Nordisk, involved 13 non‐interventional phase 4 studies in Europe (Denmark, Italy, Finland, Sweden, Switzerland, The Netherlands, Spain, and the United Kingdom), North America (Canada and Mexico), the Middle East (Saudi Arabia and Israel), and East Asia (Japan). The objective of PIONEER REAL was to evaluate the administration of oral semaglutide in standard clinical settings among adults with T2D who had not previously received injectable glucose‐lowering therapies [16]. In this study, we provide data from Mexico (ClinicalTrials.gov registration NCT04601753).

## 2 Methods

The PIONEER REAL Mexico study was a multicentre, prospective, open‐label trial conducted at 26 locations throughout Mexico. Among the treating physicians, 55.4% were endocrinologists and 44.6% were primary care doctors. The study adhered to the principles outlined in the Declaration of Helsinki and received approval from the independent ethics committee or institutional review board at each participating centre. Written informed consent was obtained from all participants prior to their enrolment in the study.

Individuals diagnosed with T2D qualified for participation if they had never used injectable glucose‐lowering treatments before and began oral semaglutide as prescribed by their physician. In addition, patients had to have an HbA1c value recorded up to 90 days before visit 1 or taken at visit 1 in accordance with local clinical practice. Blood samples were analysed in local laboratories following standard clinical practices, with HbA1c measured according to each centre's approved protocol. Participants were excluded whether they were participating in a clinical trial receiving the investigational drug ≤ 30 days prior to enrolment or in the case of mental incapacity, unwillingness, or language barriers that would interfere with the normal performance of the study. No additional diagnostic, monitoring, or therapeutic interventions were performed before enrolment. Moreover, the decisions to initiate oral semaglutide, escalate the dose, and set the maintenance dose, as well as to modify concomitant nonpharmacological and pharmacological treatments, were at the discretion of the treating physician.

At the beginning of the study (initiation of treatment, visit 1), demographic data, medical history data, and concomitant treatments for diabetes and other conditions were collected directly from the patient. Physicians also noted the reasons for starting oral semaglutide. Baseline data were obtained within 90 days preceding visit 1. Intermediate visits (visit 2.*x*) could be scheduled according to local clinical practice; the end of study visit (EOS) (visit 3) was at 38 weeks after initiation of oral semaglutide (Supplementary Figure 1).

The main endpoint was the change in HbA1c (%) from baseline to EOS. The secondary endpoints were: changes in body weight from the start to EOS (measured both as a percentage and in kilograms), changes in waist circumference (in centimetres) over the same period, the percentage of patients achieving HbA1c levels below 7%, and combined outcomes such as a reduction in HbA1c of at least 1% along with a decrease in body weight of at least 3% or 5% by the end of the study [17]. In addition, changes in satisfaction with treatment from baseline to the EOS were assessed using the Diabetes Treatment Satisfaction Questionnaire status (DTSQs). The DTSQs includes eight questions assessing diabetes treatment satisfaction on a Likert scale from 0 (very dissatisfied) to 6 (very satisfied); six items are summed for a total score [18]. Exploratory endpoints included patient continuation of oral semaglutide at EOS, daily dose at EOS, changes in other glucose‐lowering medications, incidence of self‐reported severe hypoglycaemia (needing external assistance), and physician‐assessed clinical success at EOS. Finally, adverse events (AEs) and serious AEs (SAEs) were also collected.

## 3 Statistical Analysis

Sample size was estimated using CI precision, mean change in the primary endpoint, and HbA1c change from baseline to EOS. The sample size was set to ensure a 90% chance of achieving a mean change from baseline of 95%, with a maximum half‐width of 0.30. A half‐width of 0.30 was selected to provide reliable assessment of glycaemic effectiveness consistent with diabetes guidelines [4]. The SD for mean HbA1c change was set at 1.7% based on prior studies [19, 20, 21]. Assuming that 75% of patients would have an available HbA1c measurement at end of study (EOS), it was necessary to initiate treatment with oral semaglutide in 194 patients to ensure that 145 patients had an HbA1c measurement available at EOS.

The full analysis set (FAS) for the in‐study observation period comprised all eligible patients who provided informed consent and commenced treatment with oral semaglutide. The FAS was used to characterize the study population and to perform the primary and secondary analyses, as well as to describe all reported AEs. Descriptive statistics (mean, SD for continuous variables; proportion and count for categorical variables) summarized patient characteristics at the start of oral semaglutide. Analyses were performed based on a crude and an adjusted model. A mixed model for repeated measurements (MMRM) was used to analyse the primary and secondary endpoints—HbA1c, body weight, and waist circumference. This model included adjustments for age, baseline body mass index, time, and time‐squared as covariates, while sex, the number of oral glucose‐lowering agents at baseline, diabetes duration, and study site were treated as fixed factors, with random intercepts and slopes for time. Changes in the DTSQs at the end of the study were evaluated using analysis of covariance. In addition to the primary analysis, a pattern‐mixture model was applied to all patients during the in‐study observation period. Two conditional models were specified: one representing patients who continued treatment, and another for those who discontinued treatment. This model served as a sensitivity analysis of the primary analysis. Results from statistical analyses on primary and secondary endpoints were presented, if applicable, by the estimate of the relevant clinical parameter with the associated two‐sided 95% CI. All endpoint analyses were conducted using two‐sided statistical tests with a significance threshold of 0.05. Statistical evaluations were performed utilizing SAS version 9.4 (SAS Institute, North Carolina, USA).

## 4 Results

**FIGURE 1:**
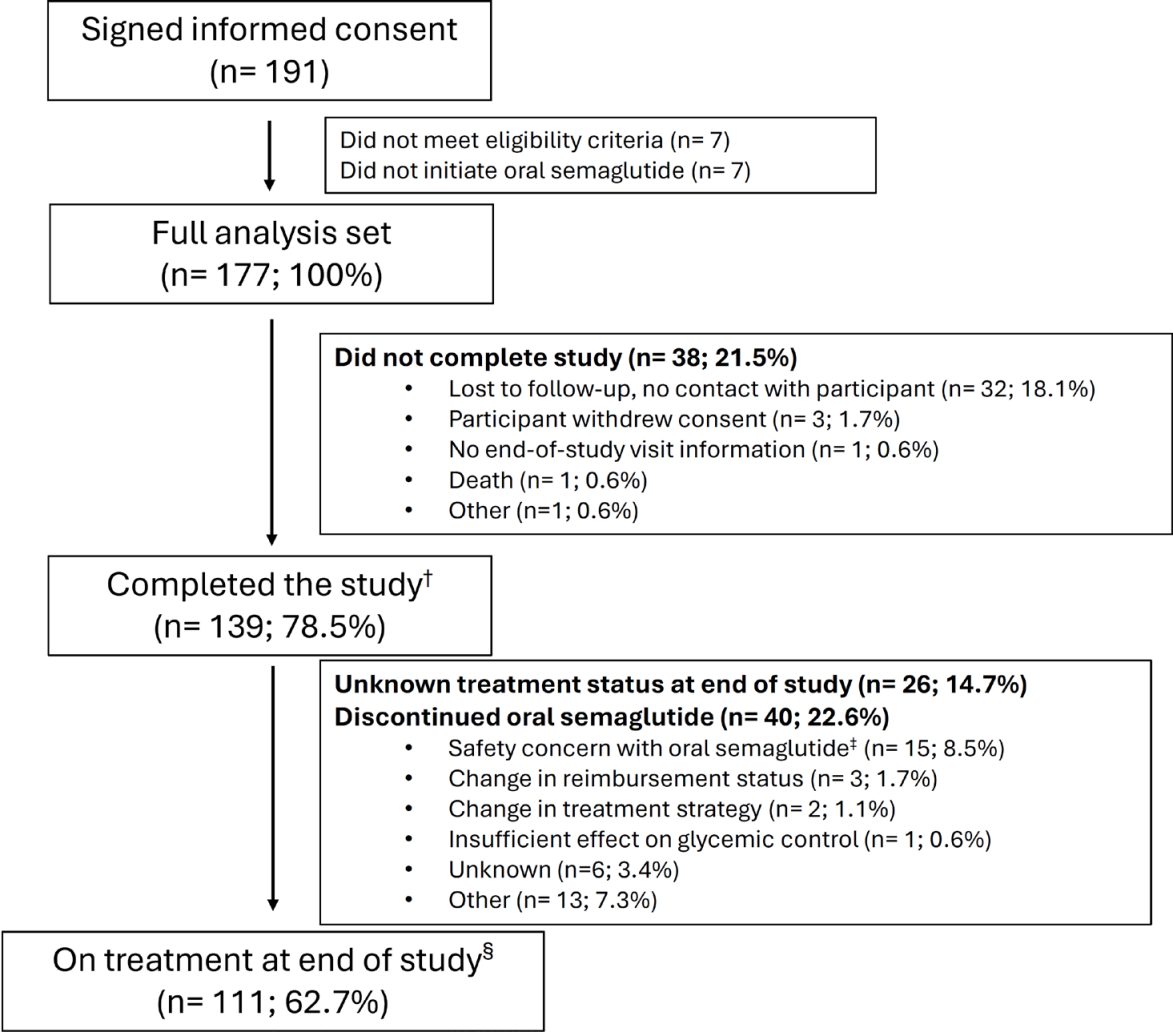
Study flow chart. ^†^Participants who initiated oral semaglutide and attended the end of study visit. ^‡^As recorded on the ‘Discontinuation of oral semaglutide form’ in the electronic case report form. ^§^Participants who were receiving oral semaglutide treatment and attended the end of study visit. Percentage values were based on the full analysis set.

Of 191 patients who signed informed consent, 7 were ineligible and 7 did not start oral semaglutide treatment. In total, 177 patients were included in the FAS. Of these, 139 patients (78.5%) finished the study. At EOS, 111 out of 177 patients (62.7%) remained on treatment (see Figure 1).

Table 1 presents the baseline clinical characteristics for the overall study population (FAS). The mean (SD) age was 54.0 (10.96) years, 57.6% of patients were women, 59.3% had obesity, mean (SD) baseline body mass index was 32.3 (5.95) kg/m^2^, and waist circumference was 104.7 (14.76) cm. Mean (SD) T2D duration was 5.7 (6.45) years; mean HbA1c was 8.1% (1.85%). Cardiovascular history, including chronic kidney disease, microalbuminuria, hemoglobinopathy and dyslipidemia, was available for 79 patients (44.6%).

**TABLE 1:** Baseline clinical characteristics (full analysis set, *N* = 177).

Variables	Value
**Biodemographic data**	
Age, *N*	177
Mean (SD), years	54.0 (10.96)
< 45 years, *n* (%)	36 (20.3)
45–65 years, *n* (%)	111 (62.7)
65–75 years, *n* (%)	28 (15.8)
> 75 years, *n* (%)	2 (1.1)
Sex (female), *N*	177
	102 (57.6)
Area of residence, *N*	174
Urban, *n* (%)	165 (94.8)
Rural, *n* (%)	9 (5.2)
Highest level of education, *N*	174
Did not attend/complete high school, *n* (%)	16 (9.2)
High school or equivalent, *n* (%)	43 (24.7)
Vocational or technical school, *n* (%)	22 (12.6)
College or university degree, *n* (%)	79 (45.4)
Postgraduate degree, *n* (%)	14 (8.0)
Occupational status, *N*	175
Work full time, *n* (%)	107 (61.1)
Work part time, *n* (%)	25 (14.3)
Student, *n* (%)	1 (0.6)
Student and work part time, *n* (%)	0
Not working (retired), *n* (%)	13 (7.4)
Not working (disabled), *n* (%)	1 (0.6)
Not working (other), *n* (%)	28 (16.0)
Reimbursement status, *N*	174
Fully covered by third party (no own co‐payment), *n* (%)	25 (14.4)
Partly covered by third party (partial own co‐payment), *n* (%)	12 (6.9)
Not covered by third party (fully paid out of pocket), *n* (%)	137 (78.7)
**Physical examination**	
Blood pressure, *N*	177
Systolic, mmHg	124.4 (12.88)
Diastolic, mmHg	77.3 (9.27)
Body mass index, *N*	177
Mean (SD), kg/m^2^	32.3 (5.95)
Underweight (< 18.5 kg/m^2^), *n* (%)	0
Normal (18.5–< 25 kg/m^2^), *n* (%)	16 (9.0)
Overweight (25–< 30 kg/m^2^), *n* (%)	56 (31.6)
Obese (≥ 30 kg/m^2^), *n* (%)	105 (59.3)
Waist circumference, *N*	175
Mean (SD), cm	104.7 (14.76)
**Diabetes parameters**	
Duration of T2D, *N*	177
Mean (SD), years	5.7 (6.45)
< 1 years, *n* (%)	51 (28.8)
1 to 5 years, *n* (%)	55 (31.1)
5 to 10 years, *n* (%)	25 (14.1)
> 10 years, *n* (%)	46 (26.0)
Microvascular complications, *N*	177
Diabetic retinopathy, *n* (%)	1 (0.6)
Diabetic neuropathy, *n* (%)	8 (4.5)
Diabetic nephropathy, *n* (%)	3 (1.7)
HbA1c level, *N*	176
< 16%, *n* (%)	176 (100)
< 14%, *n* (%)	175 (99.4)
< 12%, *n* (%)	167 (94.9)
< 10%, *n* (%)	149 (84.7)
< 8%, *n* (%)	102 (58.0)
< 7.5%, *n* (%)	< 83 (47.2)
< 7%, *n* (%)	49 (27.8)
Individualized HbA1c (%) target agreed with patient, *N*	177
Mean (SD), %	6.6 (0.56)
≤ 6.5%, *n* (%)	102 (57.6)
> 6.5 to ≤ 7.0%, *n* (%)	68 (38.4)
> 7.0 to ≤ 7.5%, *n* (%)	3 (1.7)
> 7.5 to ≤ 8.0%, *n* (%)	1 (0.6)
> 8.0%, *n* (%)	3 (1.7)
Self‐reported severe hypoglycaemic episodes at baseline*, *N*, *n* (%)	177
	0
**Comorbidities**	
Cardiovascular‐related¤ medical history, *N*	177
	53 (29.9)
Cardiovascular‐related¤ medical history including CKD, microalbuminuria, hemoglobinopathy, dyslipidemia, *N*, *n* (%)	177
	79 (44.6)
Smoking, *N*	177
Never smoked, *n* (%)	122 (68.9)
Previous smoker, *n* (%)	36 (0.3)
Current smoker, *n* (%)	19 (10.7)
**Nonpharmacological therapies**	
≥ 150 min/week of moderate physical activity, *N*, *n* (%)	177
	53 (29.9)
On a low‐calorie diet (≥ 500 kcal/day), *N*, *n* (%)	177
	47 (26.6)
**Pharmacological therapies**	
Concomitant anti‐diabetic medications at baseline, *N*	177
Metformin, *n* (%)	64 (36.2)
Sulfonylureas, *n* (%)	10 (5.6)
Alpha glucosidase inhibitors, *n* (%)	0
Thiazolidinediones, *n* (%)	3 (1.7)
Dipeptidyl peptidase 4 inhibitors, *n* (%)	7 (4.0)
Glucagon‐like peptide‐1 receptor (GLP‐1) analogues, *n* (%)	0
Sodium‐glucose co‐transporter 2 (SGLT2) inhibitors, *n* (%)	17 (9.6)
Meglitinides, *n* (%)	0
Other*, *n* (%)	2 (1.1)
No medication, *n* (%)	99 (55.9)
Concomitant fixed dose combinations at baseline	
Vildagliptin and metformin, *n* (%)	1 (0.6)
Dapagliflozin and metformin, *n* (%)	13 (7.3)
Canagliflozin and metformin, *n* (%)	1 (0.6)
Empagliflozin and metformin, *n* (%)	6 (3.4)
Concomitant cardiovascular medications at baseline, *N*	88
Diuretics, *n* (%)	20 (22.7)
Vasoprotectives, *n* (%)	3 (3.4)
Beta blockers, *n* (%)	14 (15.9)
Calcium channel blockers, *n* (%)	19 (21.6)
Renin angiotensin system inhibitors, *n* (%)	58 (65.9)
Lipid‐lowering treatment, *n* (%)	49 (55.7)
Antiplatelets, *n* (%)	8 (9.1)
Direct factor Xa inhibitors, *n* (%)	1 (1.1)
Other*, *n* (%)	1 (1.1)
**Laboratory parameters**	
Glycaemic parameters (SD)	
HbA1c (*N* = 176), %	8.1 (1.85)
HbA1c (*N* = 176), mmol/mol	65.1 (20.24)
FPG (*N* = 170), mg/dL	162.6 (67.30)
FPG (*N* = 170), mmol/L	9.0 (3.73)
**Renal parameters (SD)**	
Serum creatinine (*N* = 160), mg/dL	0.8 (0.21)
Serum creatinine (*N* = 160), μmol/L	71.3 (18.73)
eGFR (CKD‐EPI), *N* mL/min/1.73 m^2^	160
	92.8 (17.41)
< 30 mL/min/1.73 m^2^, *n* (%)	1 (0.6)
30–< 60 mL/min/1.73 m^2^, *n* (%)	9 (5.6)
60–< 90 mL/min/1.73 m^2^, *n* (%)	49 (30.6)
≥ 90 mL/min/1.73 m^2^, *n* (%)	101 (63.1)
**Lipid profile (SD)**	
Total cholesterol (*N* = 168), mg/dL	186.6 (44.26)
Total cholesterol (*N* = 168), mmol/L	4.8 (1.15)
LDL cholesterol (*N* = 152), mg/dL	111.9 (39.00)
LDL cholesterol (*N* = 152), mmol/L	2.9 (1.01)
HDL cholesterol (*N* = 154), mg/dL	44.3 (11.91)
HDL cholesterol (*N* = 154), mmol/L	1.1 (0.31)
Triglycerides (*N* = 168), mg/dL	207.6 (156.37)
Triglycerides (*N* = 168), mmol/L	2.3 (1.77)

*Note:* Cardiovascular‐related medical history includes atrial fibrillation, chronic heart failure, coronary heart disease, hypertension, peripheral artery disease, revascularization, stroke, or transient ischemic attack. Severe hypoglycaemia is defined as an episode of hypoglycaemia requiring the assistance of another person to actively administer carbohydrate or glucagon or take other corrective action.

Abbreviations: %, percentages are based on patients with responses; eGFR, estimated glomerular filtration rate; FPG, fasting plasma glucose; *N*, number of patients with response; T2D, type 2 diabetes.

* All episodes of severe hypoglycaemia occurring during the study between informed consent and treatment initiation visit (V1).

The most frequently used anti‐hyperglycaemic medications at initiation of oral semaglutide were metformin (64 patients [36.2%]), sodium‐glucose transport protein 2 (SGLT2) inhibitors (17 patients [9.6%]), and sulfonylureas (10 patients [5.6%]). A total of 99 patients (55.9%) were not receiving other anti‐hyperglycaemic medications. Of 177 patients, 175 (98.9%) initiated the 3.0‐mg dose at baseline, and the remaining two (1.1%) initiated the 7.0‐mg dose. The reason for initiating oral semaglutide included improving glycaemic control in 170 patients (96.0%), reducing body weight in 129 patients (72.9%), addressing cardiovascular risk factors in 50 patients (28.2%), and simplifying the current treatment regimen in 28 patients (15.8%). The mean (SD) actual time of exposure to oral semaglutide was 30.6 (16.39) weeks. At the EOS, the mean (SD) oral semaglutide dose was 11.5 (3.54) mg. Out of the 177 patients in the FAS, 111 (62.7%) were taking oral semaglutide at EOS. Of 111 patients on oral semaglutide at EOS, 65.8% received 14.0 mg, 31.5% received 7.0 mg, and 2.7% received 3.0 mg. Out of 177 patients, 28 (15.8%) either started a new glucose‐lowering medication or had their existing dose (excluding oral semaglutide) increased. For 17 patients (9.6%), their original glucose‐lowering medication was discontinued or its dose (again, not including oral semaglutide) was reduced during the study. Additionally, among the 177 patients for whom data were available, clinical success was attained in 98 cases (71.0%), as evaluated by their respective treating physicians.

**FIGURE 2:**
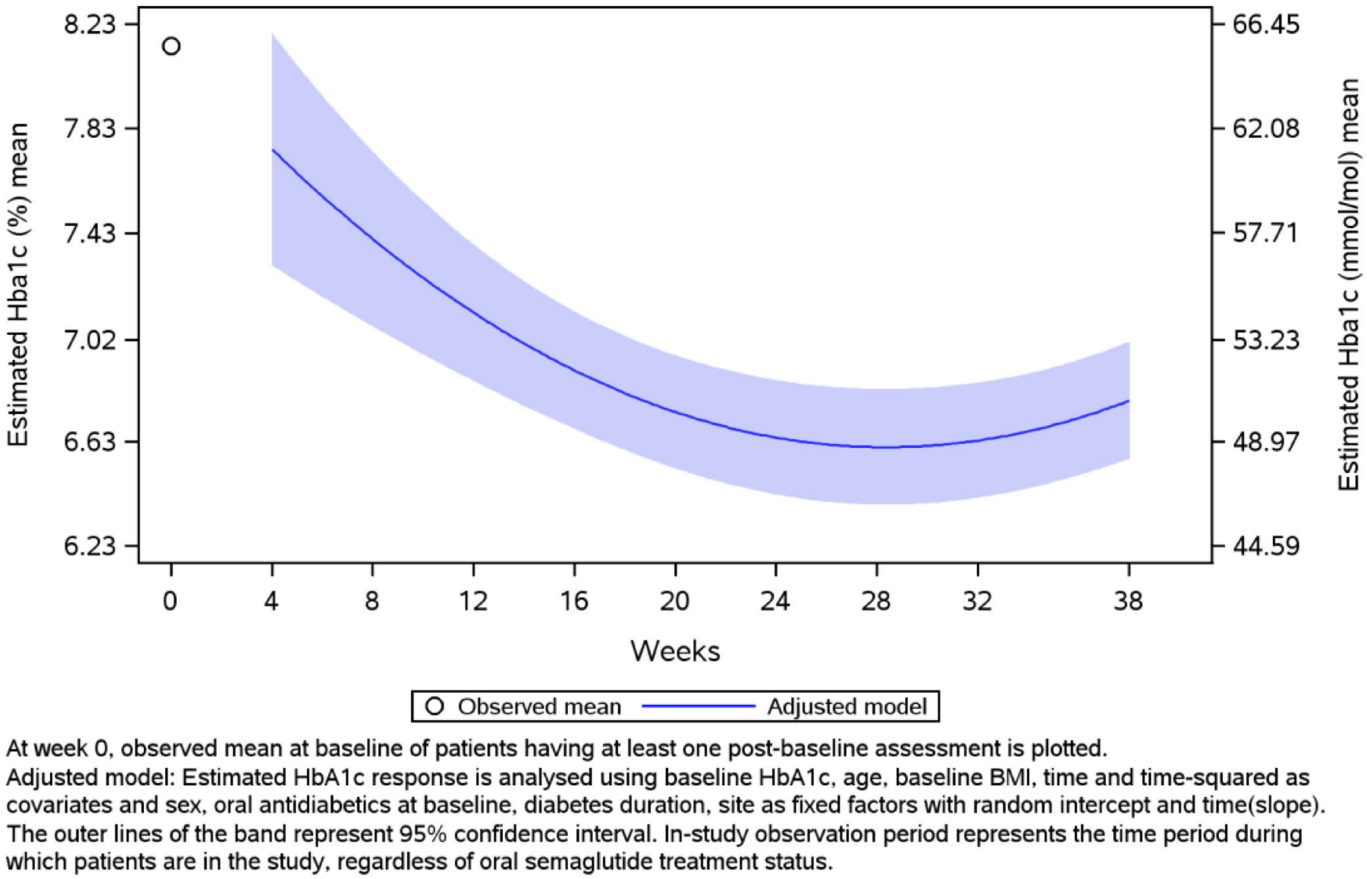
Estimated mean HbA1c over time. plot—MMRM‐adjusted—in‐study—full analysis set.

**FIGURE 3:**
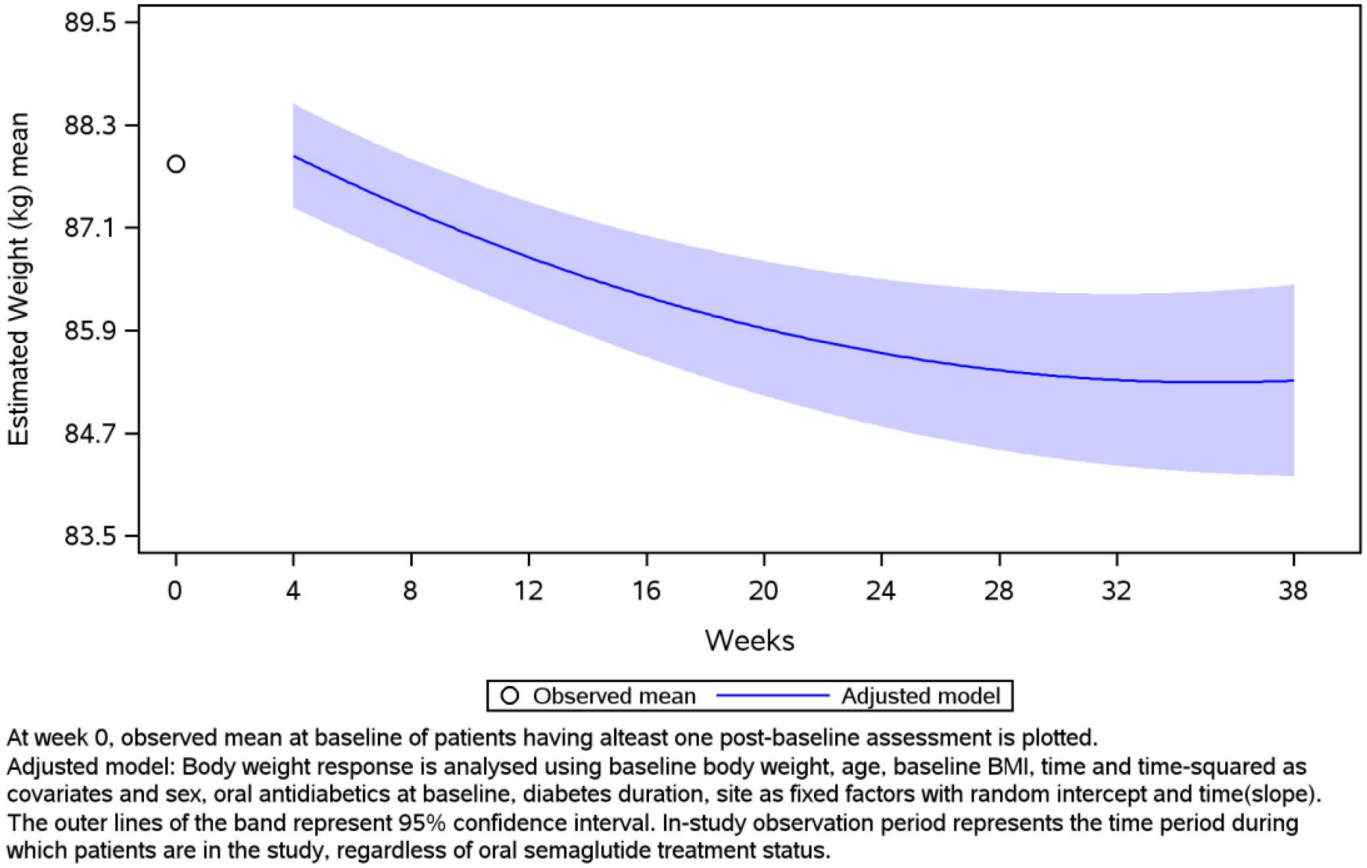
Estimated mean body weight over time. plot—MMRM‐adjusted—in‐study—full analysis set.

Regarding the primary endpoint of the study (MMRM‐adjusted), HbA1c decreased from 8.1% (1.84) to 6.8% at week 38 (estimated mean [standard error (SE)] change in HbA1c, −1.3% [0.11]; *p* < 0.0001) (Figure 2). The prespecified secondary, sensitivity, and supplementary sensitivity analyses of the primary endpoint corroborated the findings of the primary analysis (see Supplementary Figure 2). Body weight decreased from 87.8 kg (19.34) to 85.3 kg at week 38 (estimated mean [SE] change in body weight, −4.3 kg [0.56]; *p* < 0.0001) (Figure 3). The crude MMRM model for change in body weight from baseline to week 38 generated results similar to those of the adjusted model (Supplementary Figure 3). Body weight decreased by ≥ 3% from baseline to EOS in 56.3% of patients and by ≥ 5% from baseline to EOS in 42.2% (Table 2). A significant reduction in waist circumference was also observed during the study (estimated mean [SE] change of −3.7 cm [0.54]; *p* < 0.0001) (Table 2, Supplementary Figure 4). Additionally, at EOS, 60.5% of patients had attained HbA1c < 7%, 35.5% had achieved the combined goal of HbA1c reduction ≥ 1% and body weight reduction ≥ 3%, and 28.2% had reached the combined target of HbA1c reduction ≥ 1% and body weight reduction ≥ 5% (Supplementary Table 2 and Supplementary Figures 5 and 6). The observed mean (SD) DTSQs score increased from 24.7 (8.57) to 31.0 at the EOS (estimated mean [SE] change of 6.3 [0.47]; *p* < 0.0001). Similar results were obtained from the analysis of DTSQs using the crude analysis of covariance model (Table 2, Supplementary Figure 7). Changes in laboratory parameters during the study are reported in Table 3. Overall, there was a significant improvement in glycaemic parameters and lipid profile, with no significant changes in renal function.

**TABLE 2:** Change in HbA1c, body weight, waist circumference, and DTSQs from baseline to week 38.*

	Estimate	SD/SE	95% CI	*p*
HbA1c (%)				
Baseline	8.1	1.84^a^		
Week 38	6.8			
Absolute change from baseline to week 38	−1.3	0.11	−1.55; −1.10	< 0.0001
HbA1c (mmol/mol)				
Baseline	65.5	20.16^a^		
Week 38	50.7			
Absolute change from baseline to week 38	−14.5	1.24	−16.94; −12.04	< 0.0001
Body weight				
Baseline	87.8	19.34^a^		
Week 38	85.3			
Absolute change from baseline to week 38 (kg)	−4.3	0.56	−5.39; −3.15	< 0.0001
Relative change from baseline to week 38 (%)	−4.6	0.62	−5.80; −3.34	< 0.0001
Waist circumference (cm)				
Baseline	104.9	14.41^a^		
Week 38	102.6			
Absolute change from baseline to week 38 (cm)	−3.7	0.54	−4.78; −2.63	< 0.0001
DTSQs score				
Baseline	24.7	8.57^a^		
Week 38	31.0			
Absolute change from baseline to week 38	6.3	0.47	5.35; 7.22	< 0.0001

*Note:* Patients with at least one post‐baseline HbA1c/body weight/waist circumference value are included in the analysis. Estimated response and change in response are analysed from baseline to week 38 (as the EOS ranges from 34 to 52 weeks) using baseline HbA1c, age, baseline BMI, time, and time‐squared as covariates and sex, oral antidiabetics at baseline, diabetes duration, and site as fixed factors with random intercept and time (slope). Estimated response at EOS and change in response from baseline to EOS are analysed using baseline DTSQs, baseline HbA1c, age, and baseline body mass index as covariates and sex, oral antidiabetics at baseline, diabetes duration, and site as fixed factors. The *p* value is for no mean change in response. The DTSQs item score ranges from 0 (very dissatisfied) to 6 (very satisfied). Items 2 and 3 are rated as 0 (never) to 6 (most of time). All item scores, except 2 and 3, are added to a total score (range 0–36). Higher and lower DTSQs total scores indicate higher and lower treatment satisfaction, respectively. The *p* value is reported for no mean change in response.

Abbreviations: CI, confidence interval; DTSQs, Diabetes Treatment Satisfaction Questionnaire‐status; EOS, end of study; MMRM, mixed model for repeated measurements; SD, standard deviation; SE, standard error.

^a^ Standard deviation reported.

* Primary statistical analysis—MMRM‐adjusted. In‐study—full analysis set.

**TABLE 3:** Changes in laboratory parameters during the study.

Laboratory parameter (SD)	Baseline	Study end
HbA1c, %	8.1 (1.85)	6.7 (1.32)
HbA1c, mmol/mol	65.1 (20.24)	49.5 (14.46)
FPG, mg/dL	162.6 (67.30)	123.5 (37.24)
FPG, mmol/L	9.0 (3.73)	6.9 (2.07)
Serum creatinine, mg/dl	0.8 (0.21)	0.8 (0.19)
Serum creatinine, μmol/L	71.3 (18.73)	71.2 (16.94)
eGFR (CKD‐EPI), mL/min/1.73 m^2^	92.8 (17.41)	91.7 (14.61)
Total cholesterol, mg/dL	186.6 (44.26)	171.6 (41.58)
Total cholesterol, mmol/L	4.8 (1.15)	4.4 (1.08)
LDL cholesterol, mg/dL	111.9 (39.00)	100.3 (35.09)
LDL cholesterol, mmol/L	2.9 (1.01)	2.6 (0.91)
HDL cholesterol, mg/dL	44.3 (11.91)	46.3 (17.61)
HDL cholesterol, mmol/L	1.1 (0.31)	1.2 (0.46)
Triglycerides, mg/dL	207.6 (156.37)	162.2 (89.02)
Triglycerides, mmol/L	2.3 (1.77)	1.8 (1.01)

Abbreviations: eGFR, estimated glomerular filtration rate; SD, standard deviation.

Out of 211 adverse events (AEs) reported, 203 were classified as non‐serious (occurring in 81 patients: 150 mild and 53 moderate), while 8 serious adverse events (SAEs) were reported in 6 patients. In terms of causality, 87 AEs in 46 patients (26%) were reported as probably related, 56 AEs in 26 patients (14.7%) were reported as possibly related, and 68 AEs in 44 patients (24.9%) were reported as unlikely in terms of causal relationship. The most frequently reported AEs were gastrointestinal disorders (127 AEs), including nausea (40 AEs), diarrhoea (23 AEs), constipation (20 AEs), dyspepsia (13 AEs), vomiting (10 AEs), and abdominal distension (5 AEs). Seventeen AEs in 11 patients (6.2%) led to withdrawal of oral semaglutide. One episode of severe hypoglycaemia was reported in one patient (0.6%).

## 5 Discussion

The prospective real‐world PIONEER REAL Mexico study reported that oral semaglutide treatment was associated with significant reductions in HbA1c levels and body weight (decreases of 1.3% and 4.3 kg from baseline to end of study, respectively), as well as a notable improvement in treatment satisfaction. In addition, 71.0% of patients achieved clinical success, as assessed by their treating physician. These findings align with the PIONEER phase 3a trial (Supplementary Table 1) [6, 7, 8, 9, 10, 11, 12, 13] and a pooled analysis of seven country‐specific PIONEER REAL studies from Canada, Denmark, Italy, the Netherlands, Sweden, Switzerland, and the UK [16].

Type 2 diabetes has become a widespread public health concern in Mexico. This situation is expected to worsen in the coming years because of aging of the population and changes in lifestyle. In addition, glycaemic control is very far from optimal in Mexico, leading to a higher risk of complications and mortality. As a result, to reduce the burden of diabetes, it is necessary to develop health care policies that ensure a comprehensive approach to patients with T2D, including the prescription of antidiabetic drugs with proven cardiovascular benefit, like GLP‐1 RAs [22, 23, 24, 25]. In this context, real‐world studies provide relevant information that may facilitate the proper approach [14, 15].

In our study, the mean age was 54.0 years, 57.6% of patients were women, 59.3% had obesity, and nearly 45% presented with a cardiovascular‐related medical history. In the PIONEER 6 trial, the average patient age was 66 years; women comprised 32% of the cohort, and 85% of participants were aged 50 years or older with either cardiovascular disease or chronic kidney disease [11]. Therefore, relevant differences were observed in the clinical profile between patients with T2D included in the PIONEER 6 trial and our real‐world data. As a result, although this was a non‐comparative single‐arm study, our data emphasise the importance of this type of study for assessing the impact of therapy in clinical practice.

At initiation of oral semaglutide, 56% of patients in our study were not receiving other anti‐hyperglycaemic medications, 36% were taking metformin, and nearly 10% were receiving SGLT2 inhibitors. Previous studies performed in Mexico [26, 27], together with our results, clearly suggest that real‐life patients with T2D are undertreated, thus potentially explaining, at least in part, the low proportion of patients achieving HbA1c targets in clinical practice [26, 27]. Actually, nearly every time oral semaglutide was started, the main goal was to enhance glycaemic control.

At EOS, 63% of patients remained on treatment with oral semaglutide; two‐thirds were taking 14.0 mg and almost one‐third 7.0 mg. In the PIONEER 7 trial, at week 52, 59% of patients received oral semaglutide 14 mg, although 12% remained on the 3‐mg dose [12], likely due to gastrointestinal AEs that could have limited dose escalation. Moreover, in the real‐world IGNITE study, 37.0% of patients received oral semaglutide 3 mg as their highest dose, suggesting that uptitration is not performed appropriately in some patients [28]. This observation, together with the insufficient increase in use of GLP‐1 RA in clinical practice [29, 30], may reflect the potential need for education on the use of these drugs in the clinical care setting.

Regarding the primary endpoint, HbA1c decreased significantly by 1.3% at the EOS. The sensitivity analyses confirmed this result, indicating the robustness of the data. Although direct comparisons cannot be made, these data suggest that reductions in HbA1c were similar to those found in the 52‐week phase 3 PIONEER trials [7, 9, 12, 13]. Of note, whereas in phase 3 clinical trials patients were taking oral semaglutide at 14 mg, in our study, around two‐thirds were receiving 14.0 mg. As a result, the addition of oral semaglutide to patients with T2D would prove very useful for increasing the proportion of patients achieving HbA1c targets in clinical practice in Mexico, even when the 14.0‐mg dose is not attained [26, 27].

At week 38 of our study, administration of oral semaglutide resulted in a statistically significant reduction in body weight by 4.6% (equivalent to 4.3 kg) and a decrease in waist circumference by 3.7 cm. Of note, 42% of patients achieved a body weight reduction of ≥ 5% from baseline to EOS. In the phase 3 PIONEER trials, the reduction in body weight with semaglutide 14 mg was around 2.6–4.3 kg [7, 9, 12, 13]. Therefore, it seems that in clinical practice, the reduction in body weight could be even higher than in clinical trials. In fact, reducing body weight was the reason for starting oral semaglutide in 73% of our patients. Furthermore, when we analysed changes in laboratory parameters, we found a significant improvement not only in glycaemic parameters, but also in the lipid profile, with no significant changes in renal function, thus suggesting an added value in the comprehensive management of patients with T2D, as current guidelines recommend [4, 5]. In summary, the observed reductions in HbA1c, body weight, waist circumference, and improvements in lipid profiles among real‐world patients indicate that oral semaglutide supports comprehensive management of cardiovascular risk factors in individuals with T2D. These effects extend beyond glycaemic control, positioning oral semaglutide as an effective therapeutic option for managing such patients.

Satisfaction with oral semaglutide was high and increased over time. Moreover, clinical success was achieved in 71.0% of patients, as assessed by their treating physician. Overall, oral semaglutide was well tolerated, and the rate of side effects matched those found in phase 3 clinical trials [6, 7, 8, 9, 10, 11, 12, 13]. Thus, most AEs were mild to moderate in intensity, mainly involving gastrointestinal disorders, and only 17 AEs in 11 patients (6.2%) led to withdrawal of oral semaglutide. Only one episode of severe hypoglycaemia was reported (0.6%). These findings are consistent with a recent meta‐analysis indicating that combining semaglutide with basal insulin yields significant improvements in glycaemic control and reductions in body weight, without an increased risk of hypoglycaemia. This evidence supports the safety profile of semaglutide [31]. Importantly, because this was a non‐interventional study, the oral semaglutide dose depended entirely on the physician's judgement. Many patients remained on 7 or 3 mg at EOS to maintain proper glucose control, which does not necessarily mean they could not tolerate a higher dose.

This study has important strengths that highlight the significance of the results. This prospective multicentre study included 26 sites with both endocrinology and primary care, enhancing clinical representativeness. Key endpoints were HbA1c, weight, waist circumference, and DTSQs. Mixed models for repeated measures and sensitivity analyses addressed discontinuation effects, which were transparently reported (~63% remained on oral semaglutide at EOS). Safety data, primarily GI events and low severe hypoglycaemia, matched expected class effects. However, our study is subject to some limitations. Since it was a single‐arm, non‐comparative observational study, we were unable to rule out reasons other than use of oral semaglutide to explain the reduction in HbA1c and body weight. Although it would have provided valuable insights, there was no comparison of baseline characteristics between those who completed the study and those who did not. Additionally, missing data for each endpoint (HbA1c, weight, waist) were not quantified, and information regarding medication adherence was not collected. Since there was no plan to impute the missing data, there was no way to check for robustness of the data in alternative assumptions. On the other hand, since no control group was available, the relative effectiveness of semaglutide could be determined. Furthermore, concomitant antidiabetic drugs were only documented at baseline, not tracked over time, which may affect the results. However, our findings were consistent with those of previous studies, and our sensitivity analyses reinforce the validity of our results in clinical practice. Since most patients had cardiovascular disease or risk factors, our results may not apply to lower‐risk T2D subpopulations. Additionally, to establish the representativeness of the sample and its applicability to the Mexican population, it should be taken into account that the sample was predominantly urban, and most participants paid out of pocket for treatment.

In summary, our data suggests that the introduction of oral semaglutide therapy into routine clinical practice in Mexico is associated with notable decreases in HbA1c levels and body weight among patients with type 2 diabetes, as well as a significant enhancement in treatment satisfaction. Furthermore, the safety profile aligned with findings documented in prior research. Our findings suggest that oral semaglutide can be used in clinical practice as first‐line therapy in patients with T2D in Mexico to improve glycaemic control and reduce body weight as part of a comprehensive management program.

## Author Contributions

**Guillermo González‐Gálvez:** conceptualization, methodology, investigation, validation, writing – review and editing. **Aleida Y. Contreras‐Sandoval:** conceptualization, methodology, investigation, validation, writing – review and editing. **Patricia Cruz‐Puente:** conceptualization, methodology, investigation, validation, writing – review and editing. **Juan C. Garnica‐Cuellar:** conceptualization, methodology, investigation, validation, writing – review and editing. **Miguel Ángel Colín‐García:** conceptualization, methodology, investigation, validation, writing – review and editing. **Manuel Duarte‐Vega:** conceptualization, methodology, investigation, validation, writing – review and editing. **Aldo Ferreira‐Hermosillo:** conceptualization, methodology, investigation, validation, writing – review and editing. **Silvia A. Jiménez‐Ramos:** conceptualization, methodology, investigation, validation, writing – review and editing.

## Funding

Novo Nordisk provided funding for writing and editorial support. However, Novo Nordisk did not affect how data was collected, analysed or interpreted. Novo Nordisk Pharma Ltd. sponsored this study, which was registered at ClinicalTrials.gov under the identifier NCT04601753.

## Disclosure

Medical writing and editorial assistance were provided by Content Ed Net (Madrid, Spain). Editorial management was conducted by Reprints Unlimited Mexico. These services were funded by Novo Nordisk, in accordance with Good Publication Practice guidelines (www.ismpp.org/gpp‐2022).

## Ethics Statement

The research followed the guidelines of the Declaration of Helsinki and received approval from the independent ethics committee or institutional review board at each participating centre. All participants gave written informed consent before enrolment.

## Conflicts of Interest

Aleida Y. Contreras‐Sandoval and Patricia Cruz‐Puente are employees of Novo Nordisk. The rest of the authors declare not competing interests for this publication.

## Supporting information


**Supplementary Figure 1.** Study design.
Supplementary Figure 2. HbA1c (%): change from baseline to week 38.
Supplementary Figure 3. Body weight (kg and %): change from baseline to week 38*.
Supplementary Figure 4. Proportion of participants achieving body weight reduction ≥ 3%, body weight reduction ≥ 5%, and HbA1c < 7% at EOS.
Supplementary Figure 5. Proportion of participants achieving the composite endpoints of HbA1c and body weight reduction at EOS.
Supplementary Figure 6. Mean estimated waist circumference over time*.
Supplementary Figure 7. DTSQs at baseline and end of study*.
Supplementary Table 1. Change in HbA1c, body weight, and proportion of patients attaining HbA1c < 7% in the Pioneer studies.
Supplementary Table 2. Proportion of patients with a significant reduction in HbA1c and body weight.

## Data Availability

Data supporting this study's findings can be requested from the corresponding author.
