# Supplementary material for: A Multicentre, Prospective, Non‐Interventional Single‐Arm Study Investigating the Impact of Once‐Daily Oral Semaglutide in a Real‐World Adult Population With Type 2 Diabetes in Mexico
Source: Endocrinol Diabetes Metab. 2026 Apr 15;9(3):e70219. doi: 10.1002/edm2.70219 (PMC13083220; doi:10.1002/edm2.70219)
Supplement: Supplementary file 1 — Supplementary Figure 1. Study design. Supplementary Figure 2. HbA1c (%): change from baseline to week 38. Supplementary Figure 3. Body weight (kg and %): change from baseline to week 38*. Supplementary Figure 4. Proportion of participants achieving body weight reduction ≥ 3%, body weight reduction ≥ 5%, and HbA1c < 7% at EOS. Supplementary Figure 5. Proportion of participants achieving the composite endpoints of HbA1c and body weight reduction at EOS. Supplementary Figure 6. Mean estimated waist circumference over time*. Supplementary Figure 7. DTSQs at baseline and end of study*. Supplementary Table 1. Change in HbA1c, body weight, and proportion of patients attaining HbA1c < 7% in the Pioneer studies. Supplementary Table 2. Proportion of patients with a significant reduction in HbA1c and body weight. [file EDM2-9-e70219-s001.docx]

**A multicenter, prospective, non-interventional single-arm study investigating the impact of once-daily oral semaglutide in a real-world adult population with type 2 diabetes in Mexico**

**Supplementary Figures and Tables**

**Supplementary figure 1. Study design.**

**
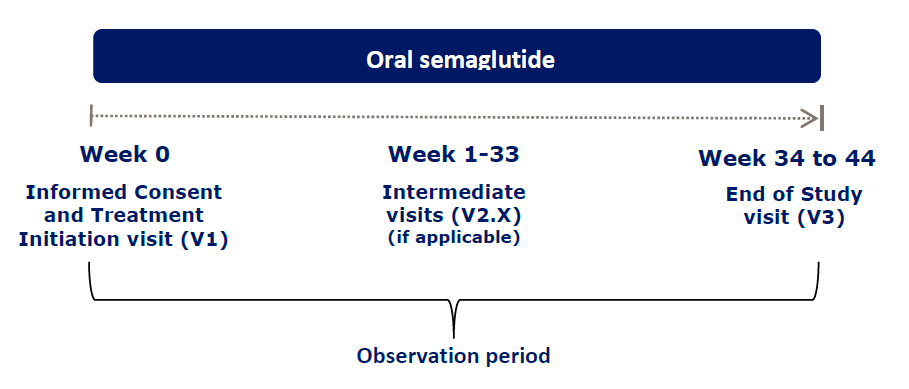
**

V = visit; V2.X = intermediate visits

**Supplementary figure 2. HbA1c (%): change from baseline to week 38*.**

**
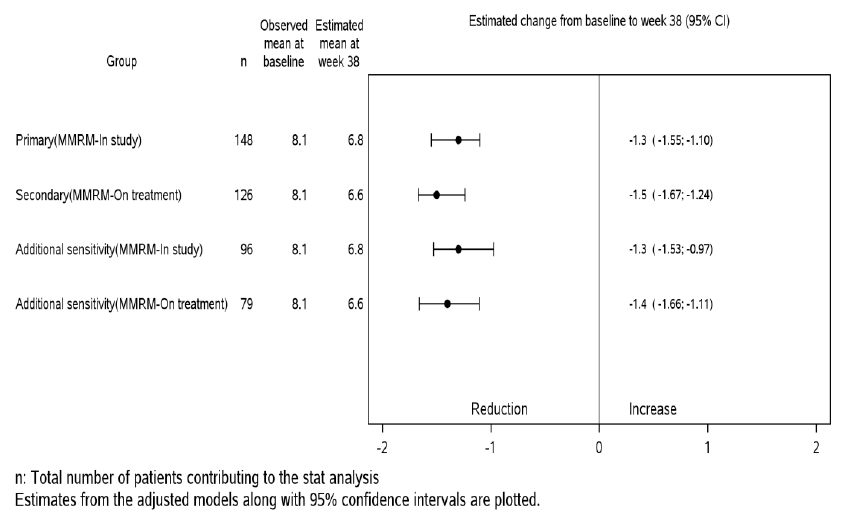
**

*- forest plot - statistical analysis - MMRM-adjusted - full analysis set.

**Supplementary figure 3. Body weight (kg and %): change from baseline to week 38*.**


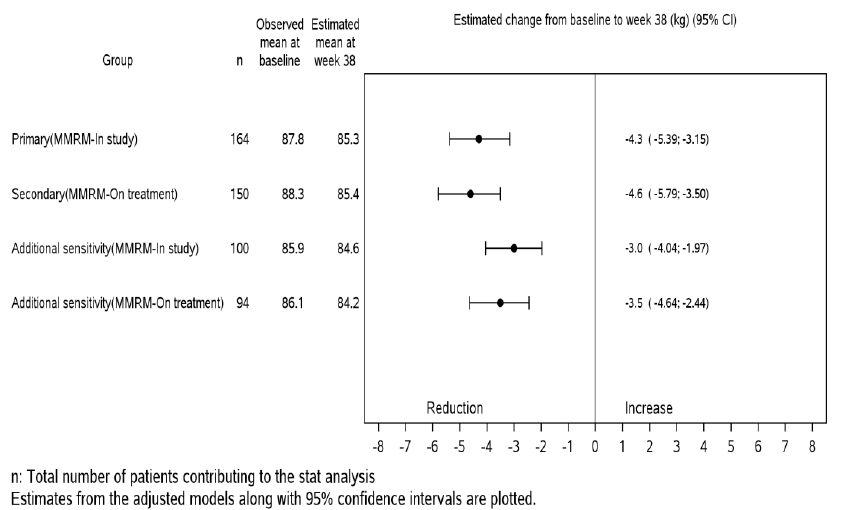


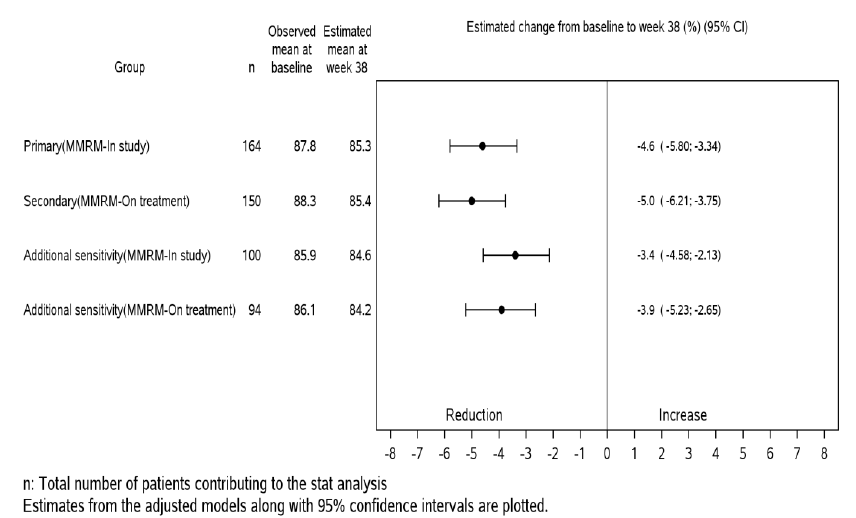


*- forest plot - statistical analysis - MMRM-adjusted - full analysis set.

**Supplementary figure 4.** **Proportion of participants achieving body weight reduction ≥3%, body weight reduction ≥5%, and HbA1c <7% at EOS.**

**
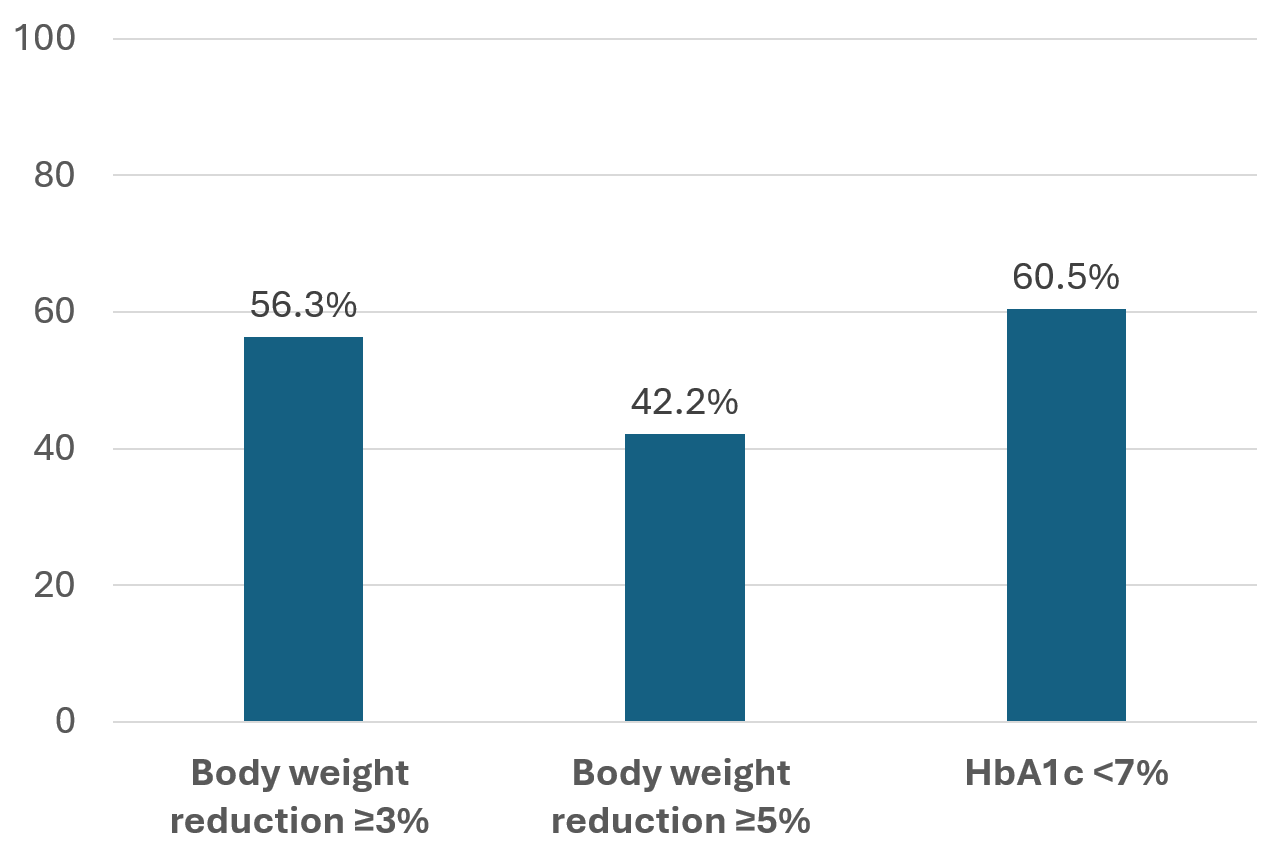
**

EOS: end of study visit; HbA1c glycated hemoglobin.

**Supplementary figure 5.** **Proportion of participants achieving the composite endpoints of HbA1c and body weight reduction at EOS.**

**
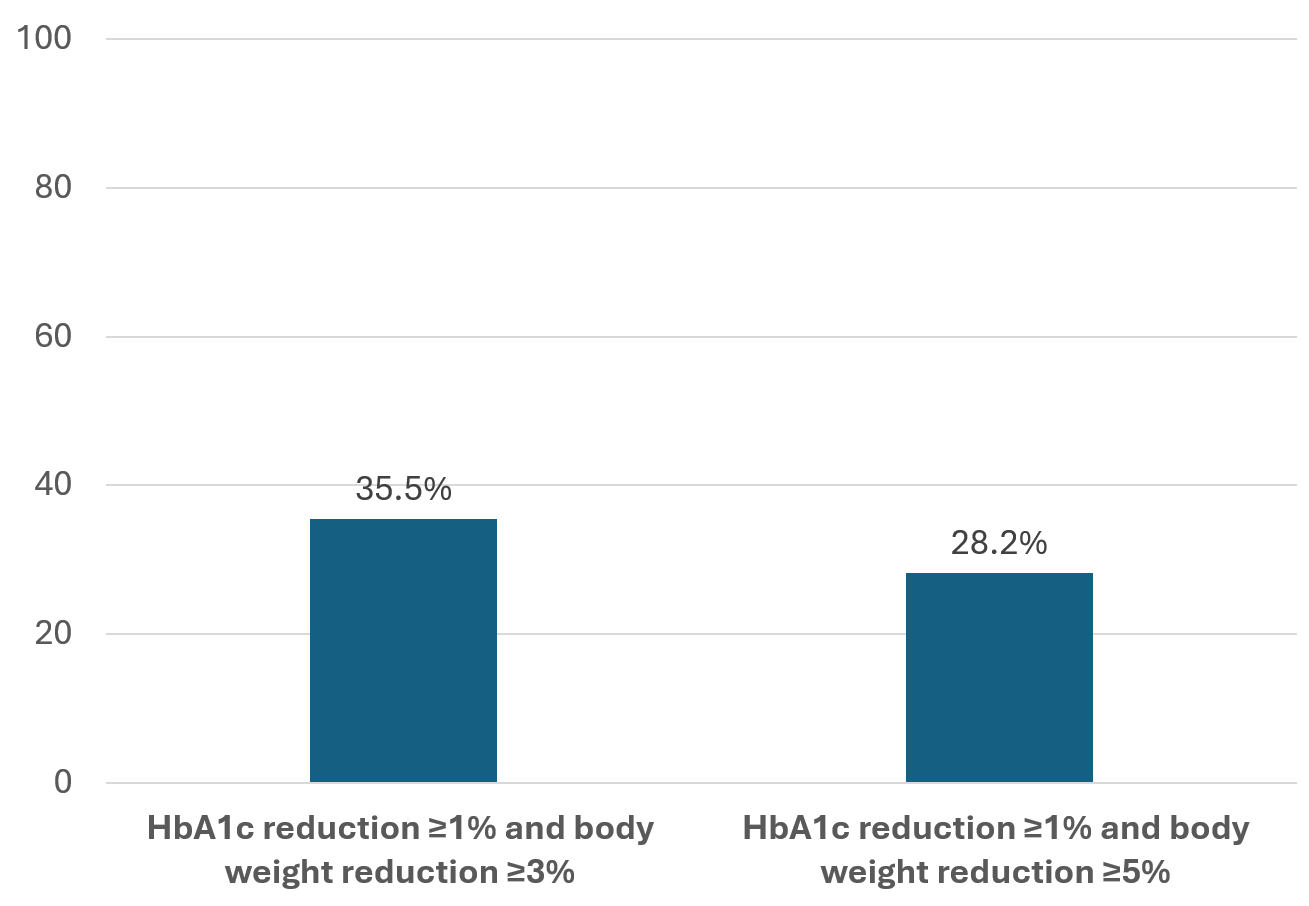
**

EOS: end of study visit; HbA1c glycated hemoglobin.

**Supplementary figure 6. Mean estimated waist circumference over time*.**

**
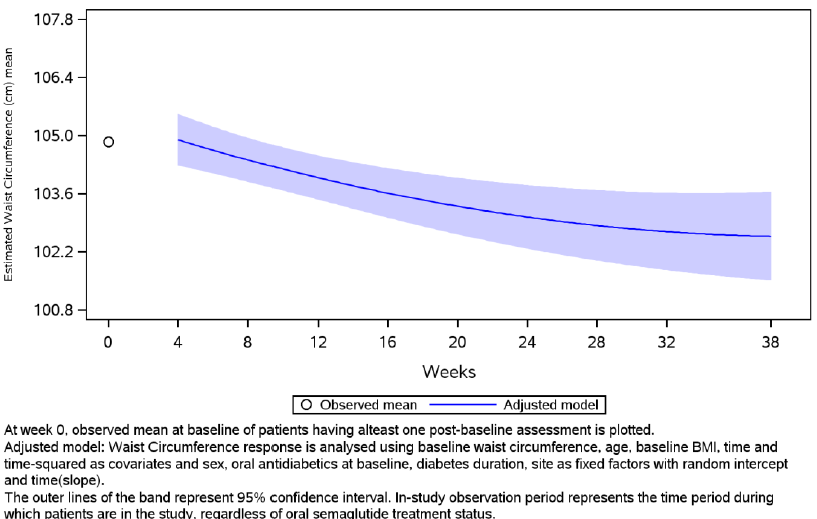
**

* - plot - MMRM-adjusted - in-study - full analysis set.

**Supplementary figure 7. DTSQs at baseline and end of study*.**

**
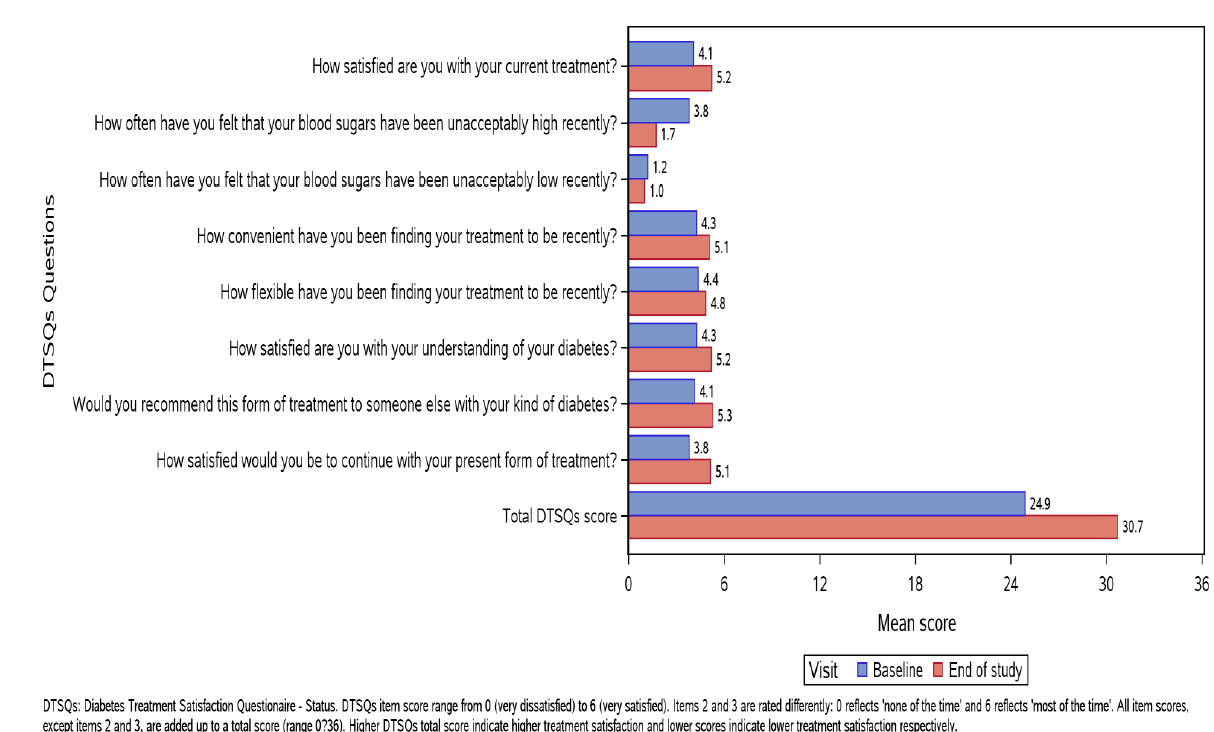
**

*- bar plot - full analysis set.

**Supplementary table 1. Change in HbA1c, body weight, and proportion of patients attaining HbA1c <7% in the Pioneer studies.**

|  |  | **Pioneer 1** | **Pioneer 2** | **Pioneer 3** | **Pioneer 4** | **Pioneer 5** | **Pioneer 6** | **Pioneer 7** | **Pioneer 8** | **Pioneer REAL** |
| --- | --- | --- | --- | --- | --- | --- | --- | --- | --- | --- |
|  |  | **Monotherapy**  **(diet and exercise)**  **26 weeks** | **vs empaglifozin**  **52 weeks** | **vs sitagliptin**  **78 weeks** | **vs liraglutide 1.8 mg**  **52 weeks** | **Moderate chronic kidney disease****  **26 weeks** | **vs placebo**  **Flexible dose adjustment**  **69 weeks** | **vs sitagliptin**  **Flexible dose adjustment**  **52 weeks** | **with insulin**  **52 weeks** | **Mexico** |
| **Baseline HbA1c%** | | 8.0 | 8.1 | 8.3 | 8.0 | 8.0 |  | 8.3 | 8.2 | 8.1 |
| **Change in HbA1, %** | **Oral semaglutide 14 mg** | -1.4* | -1.3* | -1.1* | -1.2* | -1.0* | -1.0* | -1.3* | -1.2* | -1.3 |
|  | **Placebo or comparator** | -0.3 | -0.9 | -0.7 | -0.9 | -0.2 | -0.3 | -0.8 | -0.2 | NA |
| **Change in body weight, kg** | **Oral semaglutide 14 mg** | -3.7* | -3.8 | -3.2* | -4.3* | -3.4* | -4.2* | -2.6* | -3.7* | -4.3 |
|  | **Placebo or comparator** | -1.4 | -3.6 | -1.1 | -3.0 | -0.9 | -0.8 | -0.7 | 0.5 | NA |
| **Proportion of subjects HbA1c <7%, %** | **Oral semaglutide 14 mg** | 76.9* | 66.1* | 44.0* | 55* | 58* |  | 58* | 54.2* | 60.5 |
|  | **Placebo or comparator** | 31.0 | 43.2 | 29.0 | 15.0 | 23* |  | 25* | 9.3 | NA |

*Statistically significant vs placebo or active comparator. ** Added to basal insulin, metformin, and basal insulin or metformin and/or sulfonylureas. Data from references #6-13.

**Supplementary table 2. Proportion of patients with a significant reduction in HbA1c and body weight.**

|  | **Total**  **N (%)** |
| --- | --- |
| Number of patients | 177 |
| HbA1c reduction ≥1% from baseline to EOS, N | 129  66 (51.2) |
| Body weight reduction of ≥5% from baseline to EOS, N | 128  54 (42.2) |
| Body weight reduction of ≥3% from baseline to EOS, N | 128  72 (56.3) |
| HbA1c level at EOS < 7.0% (53 mmol/mol), N | 129  78 (60.5) |
| HbA1c reduction ≥1% and body weight reduction ≥3% from baseline to EOS, N | 124  44 (35.5) |
| HbA1c reduction ≥1% and body weight reduction ≥5% from baseline to EOS, N | 124  35 (28.2) |

N: Number of patients with response, %: Percentage of patients; EOS: end of study visit.
